# The Neuroprotective Disease-Modifying Potential of Psychotropics in Parkinson's Disease

**DOI:** 10.1155/2012/753548

**Published:** 2011-12-27

**Authors:** Edward C. Lauterbach, Leonardo F. Fontenelle, Antonio L. Teixeira

**Affiliations:** ^1^Department of Psychiatry and Behavioral Sciences and the Department of Internal Medicine, Neurology Section, Mercer University School of Medicine, 1400 Coleman Avenue, Macon, GA 31201, USA; ^2^Anxiety and Depression Research Program, Institute of Psychiatry, Federal University of Rio de Janeiro (IPUB/UFRJ), CEP: 22290-140 Rio de Janeiro, RJ, Brazil; ^3^Neurology Group, Department of Internal Medicine, School of Medicine, Federal University of Minas Gerais, Belo Horizonte, MG, Brazil

## Abstract

Neuroprotective treatments in Parkinson's disease (PD) have remained elusive. Psychotropics are commonly prescribed in PD without regard to their pathobiological effects. The authors investigated the effects of psychotropics on pathobiological proteins, proteasomal activity, mitochondrial functions, apoptosis, neuroinflammation, trophic factors, stem cells, and neurogenesis. Only findings replicated in at least 2 studies were considered for these actions. Additionally, PD-related gene transcription, animal model, and human neuroprotective clinical trial data were reviewed. Results indicate that, from a PD pathobiology perspective, the safest drugs (i.e., drugs least likely to promote cellular neurodegenerative mechanisms balanced against their likelihood of promoting neuroprotective mechanisms) include pramipexole, valproate, lithium, desipramine, escitalopram, and dextromethorphan. Fluoxetine favorably affects transcription of multiple genes (e.g., MAPT, GBA, CCDC62, HIP1R), although it and desipramine reduced MPTP mouse survival. Haloperidol is best avoided. The most promising neuroprotective investigative priorities will involve disease-modifying trials of the safest agents alone or in combination to capture salutary effects on H3 histone deacetylase, gene transcription, glycogen synthase kinase-3, *α*-synuclein, reactive oxygen species (ROS), reactive nitrogen species (RNS), apoptosis, inflammation, and trophic factors including GDNF and BDNF.

## 1. Introduction

 Parkinson's disease (PD) and other neurodegenerative diseases are common and impose substantial morbidities and costs on patients, caregivers, and society [1–3]. Neuropsychiatric conditions occur in most patients with Parkinson's disease (PD), with 61–88% of patients reporting at least one psychiatric symptom [2]. Neuropsychiatric disorders include a variety of cognitive concerns, delirium, dementia, depression, anxiety, panic, and other conditions related either to PD itself or its treatment [2]. These neuropsychiatric morbidities are quite significant, with cognitive impairment and depression constituting two of the strongest determinants of PD quality of life [2]. As such, these conditions necessitate treatment.

Psychotropics are commonly used to treat these PD co-morbidities without regard to their potential pathobiological effects [3, 4]. Furthermore, psychotropics are used in treating the dementias that attend PD (Parkinson's disease dementia, dementia with Lewy bodies, and Alzheimer's disease), eventually present in nearly all patients [1, 2]. Additionally, dopaminergic therapies (levodopa, dopamine agonists) and deep brain stimulation are associated with treatment complications including mania, gambling, hypersexuality, other impulse control disorders, and suicide attempts [2]. Psychotropics are widely prescribed for these conditions, again without considering their potential disease-modifying effects.

**Figure 1:**
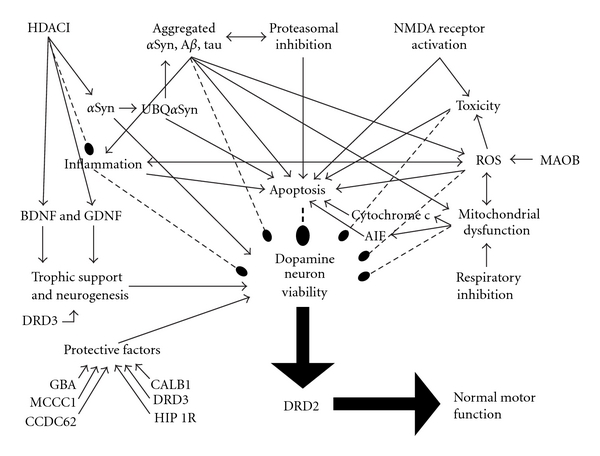
Factors affecting the viability of dopamine neurons. Relations terminating in an arrowhead indicate facilitation, those with double arrowheads indicate mutual facilitation, and dashed lines terminating in a bulb indicate inhibition. Though still being settled, recent data suggest that alpha-synuclein (*α*Syn) is neuroprotective whereas monoubiquitylated *α*Syn, aggregated *α*Syn, and other pathogenic proteins promote neurodegeneration. H3 histone deacetylase inhibition (HDACI) increases *α*Syn, brain derived neurotrophic factor (BDNF), and glial derived neurotrophic factor (GDNF), supporting neuronal synapses (*α*Syn) and providing trophic support for neurons and promoting neurogenesis (BDNF and GDNF). Neurotrophism appears to be facilitated by D3 dopamine receptor stimulation. HDACI also inhibits inflammation. Aggregated proteins inhibit the proteasome, promote reactive oxygen species (ROS), mitochondrial dysfunction, inflammation, and apoptosis, and impair neuronal viability. Inhibition of the proteasome results in reduced elimination of obsolete proteins, increases in aggregated protein species, facilitates apoptosis, and impairs neuronal viability. N-methyl-D-aspartate (NMDA) receptor activation by glutamate promotes neurotoxicity and apoptosis. Generation of peroxide radicals by MAOB promotes ROS. ROS and inflammation mutually promote each other, and each can induce apoptosis. Mitochondrial dysfunction and ROS also mutually promote each other. Impaired mitochondrial respiration through inhibition of respiratory chain complexes (I-IV) can produce mitochondrial dysfunction. Mitochondrial dysfunction leads to the loss of the mitochondrial membrane potential, opening of the mitochondrial permeability transition pore, and the release of cytochrome c and apoptosis inhibiting factor (AIF). Cytochrome c and AIF each independently trigger apoptosis. Protective factors against neurodegeneration include GBA, DRD3, CALB1, and other gene products. Thus, neurodegenerative processes include pathogenic proteins, proteasomal dysfunction, glutamate and other toxic molecules, NMDA receptor activation, ROS, mitochondrial dysfunction, apoptotic pathway activation, and subsequent neuroinflammation, in turn potentially inducing further ROS and apoptosis. Neuroprotective factors include GBA, MCCC1, CCDC62, HIP1R, DRD3, CALB1, *α*Syn, HDACI, BDNF, and GDNF. Neuroprotective factors promote while neurodegenerative processes impair the viability of the dopamine neuron. Nigral dopamine neurons promote normal motor functioning by release of dopamine on striatal D2 receptors, transcribed from the DRD2 gene, and reduced D2 stimulation is associated with Parkinson motor features.

**Figure 2:**
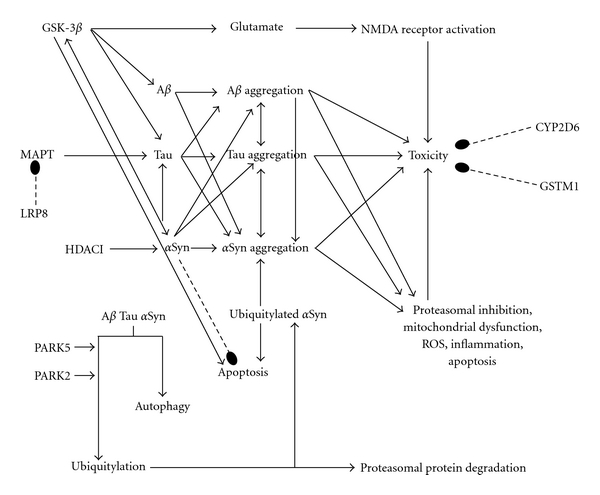
Interactions of neuroprotective and neurodegenerative pathways emphasizing pathogenic proteins and toxins. Relations terminating in an arrowhead indicate facilitation, those with double arrowheads indicate mutual facilitation, whereas dashed lines terminating in a bulb indicate inhibition. The enzyme glycogen synthase kinase 3 beta (GSK-3*β*) activates glutamatergic excitotoxicity mediated through the N-methyl-D-aspartate (NMDA) receptor. GSK-3*β* also drives production of alpha-synuclein (*α*Syn), the pathogenic proteins beta-amyloid (A*β*) and tau, and apoptosis. Whereas *α*Syn appears to be neuroprotective and inhibits apoptosis, mono-ubiquitylated *α*Syn promotes *α*Syn aggregation and apoptosis. On the other hand, *α*Syn can also increase GSK-3*β* and tau concentrations, in turn increasing aggregated *α*Syn, A*β*, and tau itself. Tau can further increase concentrations of *α*Syn. Aggregated *α*Syn, A*β*, and tau inhibit the proteasome and induce cellular toxicity, reactive oxygen species (ROS), mitochondrial dysfunction, apoptosis, and inflammation, leading to neuronal demise. The three proteins promote the formation of each other, as do their aggregated forms. The LRP8 gene product stabilizes microtubule associated protein tau (MAPT), the gene that produces tau protein, and dysfunctional LRP8 leads to excessive MAPT expression, increasing tau and driving pathogenic protein aggregation. Pathogenic proteins are disposed of through autophagy and the ubiquitin-proteasomal system, wherein proteins targeted for destruction are polyubiquitylated, a process that appears to be regulated by PARK5 (UCHL1) and PARK2 (parkin). Interference with autophagy or ubiquitylation prevents disposal of proteins, leading to their accumulation and their subsequent inhibition of the proteasome. GSTM1 and CYP2D6 gene products promote solvent detoxification, and deficiencies in these proteins permit toxicity. GSTM1 is particularly important in the context of CYP2D6 dysfunction.

Psychotropics can directly [3] and indirectly [4] affect neurodegenerative pathobiology in a variety of ways [3, 4]. For example, a drug can directly affect apoptotic mechanisms and/or can indirectly affect apoptosis by its direct effects on pathogenic proteins, the proteasome, mitochondria, free radical formation, microglial activation, or inflammation [4]. Previous work had considered the effects of psychotropics on *intracellular *processes including proteins, proteasome, mitochondrion, and apoptosis [3], supplemented by a wider array of *extracellular* actions including neuroinflammation, trophic factors, neural and glial stem cells, and neurogenesis [4] across various cell types and models [3, 4]. The potential to modify the course of a neurodegenerative disease through these effects holds substantial implications for both PD patients and society as a whole [3, 5]. Figures 1 and 2 depict the relations and interrelations of these pathobiological mechanisms in regard to the viability of dopamine neurons.

Psychotropics may also affect the transcription of genes relevant to PD. The authors were therefore interested in exploring the effects of psychotropic drugs on each of these intracellular domains in published medical literature and gene expression databases. In this paper, we provide an update focusing on neuronal neurodegenerative mechanism findings that have been replicated in mature neural tissues or demonstrated in disease-relevant animal models. Second, a survey of psychotropic effects on the mRNA expression of genes relevant to PD risk and pathobiology was undertaken. Since genetic studies have revealed genes associated with PD risk and certain mutations are associated with various PD phenotypes, the ability of psychotropics to affect gene expression could potentially modify the course of PD with either deleterious or therapeutic potential. Third and finally, we consider the extant clinical trial literature as it pertains to first-line psychotropics and neuroprotection in PD.

## 2. Materials and Methods

### 2.1. Gene Expression Search

We comprehensively surveyed gene expression as a function of psychotropic treatment for genes associated with PD risk [6] and the classical PARK1-13 mutations associated with PD [7] and PARK14–16 by assessing literature in the PubMed (http://www.ncbi.nlm.nih.gov/pubmed/) and Gene Expression Omnibus Profiles (GEO Profiles http://www.ncbi.nlm.nih.gov/sites/entrez?db=geo) databases. Risk-associated genes consisted of the genes most strongly associated with PD according to the PDGene database [6]. These genes were SNCA, MAPT/STH, NUCKS1, PM20D1, SLC41A1, BST1, LRRK2, USP24, SLC6A3, GBA, SLC45A3, SOD2, MTHFR, PLEKHM1, DGKQ, BDNF, PDXK, GWA 7p14.2, APOE, DRD3, GWA 2q36.3, GSTM1, PINK1, FGF20, CYP2D6, PARK2 (parkin), HLA-DRA, GLIS1, MAOB, CALB1, FARP1, LRP8, DRD2, UCHL1, GAK, MCCC1/LAMP3, STK39, SYT11, HLA-DRB5, CCDC62/HIP1R, ACMSD, and MED13. PARK 1–16 genes were also surveyed. 

Drugs considered included first-line direct-acting D2/D3 dopamine agonists, antipsychotics, mood stabilizers, antidepressants, anxiolytics, and dextromethorphan combined with quinidine. While a primary treatment for the underlying disease, D2/D3 dopamine agonists are also used to treat apathy and have antidepressant qualities and were therefore included in this paper. Drugs were searched by their psychopharmacological category and by their specific names in each database. Specific drug search terms used were “neuroleptic OR atypical antipsychotic OR antipsychotic OR anxiolytic OR benzodiazepine OR antidepressant OR tricyclic antidepressant OR heterocyclic antidepressant OR SSRI OR selective serotonin reuptake inhibitor OR pramipexole OR ropinirole OR amantadine OR haloperidol OR fluphenazine OR trifluoperazine OR thiothixene OR chlorpromazine OR thioridazine OR risperidone OR olanzapine OR quetiapine OR ziprasidone OR aripiprazole OR clozapine OR paliperidone OR iloperidone OR asenapine OR tetrabenazine OR pimavanserin OR lithium OR carbamazepine OR oxcarbazepine OR valproate OR lamotrigine OR amitriptyline OR imipramine OR nortriptyline OR desipramine OR clomipramine OR trimipramine OR doxepin OR protriptyline OR maprotiline OR bupropion OR fluoxetine OR sertraline OR fluvoxamine OR paroxetine OR citalopram OR s-citalopram OR trazodone OR nefazodone OR venlafaxine OR duloxetine OR mirtazapine OR atomoxetine OR buspirone OR diazepam OR chlordiazepoxide OR flurazepam OR temazepam OR clorazepate OR clonazepam OR lorazepam OR oxazepam OR alprazolam OR zaleplon OR zolpidem OR zopiclone OR s-zopiclone OR cyproheptadine OR hydroxyzine OR diphenhydramine OR benztropine OR trihexyphenidyl OR modafinil OR ramelteon OR dextromethorphan OR quinidine.” Levodopa was not reviewed because it generally is not prescribed to treat behavioral problems. Cognitive enhancers (cholinesterase inhibitors and NMDA antagonists) are not reviewed here because they have an extensive literature that has been reviewed elsewhere. The term “AND (mRNA OR gene expression)” was added to gene names, symbols, aliases, and drug terms in PubMed. A variety of models and treatment durations were encountered. Because psychotropics tend to be administered chronically in the clinical treatment of PD, only reports of chronic administration (at least 3-week duration in animal studies) are considered here.

Gene expression data in GEO Profiles was considered if a given treatment was compared to untreated controls under the same experimental conditions and if the data involved at least 2 determinations at a single locus (solitary determinations can be unreliable). Gene expression data in GEO Profiles were found for the selective serotonin reuptake inhibitor (SSRI) antidepressant fluoxetine, the neuroleptic antipsychotic haloperidol, and the atypical antipsychotics olanzapine and clozapine. Fluoxetine was administered for 21 days in mice, and gene expression was determined relative to untreated controls in the hippocampus (GEO Profiles accession number GDS2803) using the Affymetrix Gene Chip^R^ Mouse Genome 430 2.0 Array [8]. Olanzapine was given for 21 days in rats, and gene expression was compared to untreated controls in the frontal cortex (accession number GDS2608) using the Affymetrix Gene Chip^R^ Rat Genome 230 2.0 Array [9]. Results for haloperidol and clozapine relative to untreated control mice reflect gene expression in brain after treatment for 4 weeks (accession number GDS2537) using the Affymetrix Gene Chip^R^ Murine Genome U74 Version 2 Array [MG_U74Av2] and 12 weeks (accession number GDS2531) using the Affymetrix Gene Chip^R^ Mouse Expression 430A Array [MOE430A]. Investigations were limited to murine species in GEO Profiles, and no studies of gene expression in the substantia nigra or striatum were encountered.

Reporting of GEO Profiles findings is limited to genes where specific probe sets were upregulated or downregulated by at least 20%. Percentage change for a given reporter probe set was calculated as the difference of the reporter probe value for treated animals from its untreated control values divided by that control value. In cases where there were positive findings for any gene probe set, probe sets were assessed to determine their reliability in assaying gene expression. Results are provided for genes for which changes in expression were observed after considering probe set reliability. Normalized expression data in GEO Profiles were derived from a gene chip and remain to be confirmed by quantitative real-time polymerized chain reaction (RT-PCR) or other analyses.

### 2.2. Posttranscriptional Neurodegenerative Mechanisms Search

Relevant studies were identified through a literature search of *intracellular* and *extracellular *neurodegenerative mechanisms (PubMed search terms: (alpha-synuclein OR beta-amyloid OR tau OR TDP-43 OR ubiquitin OR proteasome OR mitochondrial viability OR mitochondria OR mitochondrial transition pore OR cytochrome c release OR endosome OR lysosome OR autophagy OR endoplasmic reticulum OR leukocyte viability OR apoptosis OR inflammation OR trophins OR neurogenesis OR BDNF OR GDNF OR neural stem cells) AND (neuron OR neuronal OR neurons OR glia OR glial OR neuroglia)). These terms were joined by the operator “AND” to the drug search terms detailed in the gene expression section, except that only the specific drugs listed were searched (the pharmacological class search terms were omitted, specifically “neuroleptic OR atypical antipsychotic OR antipsychotic OR antidepressant OR anxiolytic OR benzodiazepine OR antidepressant OR tricyclic antidepressant OR heterocyclic antidepressant OR SSRI OR selective serotonin reuptake inhibitor”).

Citations were reviewed with an exclusive focus on mature neural tissues because nonneural, immature neural tissues and malignancy-related cells lines have been demonstrated to behave differently with regard to the processes studied here. The sole exception occurred in disease-specific animal models, where stem cells in mature brain were also considered. We considered studies of any methodology but included only models relevant in PD (including PD-specific models and results involving cells or biological processes specifically relevant to PD). Thus, cell culture conditions not typical of PD (e.g., hyperosmotic stress, oxygen deprivation, potassium deprivation, etc.) were also excluded. 

We focused on the intracellular processes of interest as specified in the search terms and did not consider studies of other mechanisms unless those studies also considered the targeted processes. For example, intracellular calcium influx and other disease mechanisms were not examined unless they also involved the drugs and processes of interest. Deoxyribonucleic acid (DNA) fragmentation and condensation were required to ascertain apoptosis, and other indices (apoptotic mediator concentration, cell viability) were considered insufficient.

In contrast, in disease-specific animal models, outcomes consistent with putative neuroprotection were considered even if the study did not specifically address the intracellular targets required for cell and tissue studies, provided that the outcomes were relevant to PD-specific clinical outcomes. We considered studies in rotenone, 1-methyl-4-phenyl-1,2,3,6-tetrahydropyridine (MPTP), lipopolysaccharide (LPS), and other rodent PD models, a transgenic mouse model relevant to tauopathic parkinsonism (FTDP-17, or frontotemporal dementia with parkinsonism related to tau mutations on chromosome 17), and an amyotrophic lateral sclerosis (ALS) mouse model that examined alpha-synuclein (*α*Syn).

### 2.3. Neuroprotective Clinical Trials Search

Clinical studies potentially relevant to determining disease-modifying neuroprotection of drugs in PD, especially those employing neurodegenerative laboratory measures, representing first-line psychotropics were identified through a literature search and bibliographic extension across the literature (PubMed search terms: (neuroprotection OR neuroprotective OR disease modifying OR disease modifying OR disease modification OR progression OR disease progression OR biomarker OR alpha-synuclein OR cerebrospinal fluid OR imaging OR magnetic resonance imaging OR single photon emission computed tomography OR positron emission tomography) AND Parkinson's disease). These terms were joined by the operator “AND” to the drug search terms specified in the section “Posttranscriptional Neurodegenerative Mechanisms Search.” 

## 3. Results and Discussion

Chronic psychotropic treatment had some noteworthy effects on the mRNA transcription of PD risk-related genes. For the sake of brevity, gene mRNA expression findings mentioned below generally do not include negative chronic (i.e., at least 3 weeks) treatment studies unless the negative finding is specifically pertinent to the nigrostriatal tract. 

Several genes are highly associated with risk (*P* < 0.000001), mentioned here in order of strongest to weakest association with PD risk. It is important to keep in mind that the rank ordering of associated genes can change over time as more data are reported. MAPT (this gene's official designation) is located at q21.1 on chromosome 17 (17q21.1) and is the gene for microtubule-associated protein tau. It has the strongest association with PD risk of all genes. Increased promoter region function, especially with the H1 haplotype, is associated with late-onset PD. Tau and *α*Syn proteins interact to mutually promote their synthesis and aggregation (see Lithium and Valproate sections below). GBA encodes for acid beta-glucosidase (1q21), mutations of which are linked to PD through an obscure mechanism. MCCC1 (3q27) is the gene for methylcrotonoyl-coenzyme A carboxylase 1 alpha. This protein is involved in nucleotide binding, catalytic activity, ATP binding, biotin binding, and ligase activity and is found in Golgi apparatus and the mitochondrial matrix and inner membrane. Deficiency impairs leucine degradation and produces an organic acidemia with neurological features. CCDC62/HIP1R (12q24.31/12q24) involves two different genes. Coiled-coil domain containing 62 (CCDC62) is involved with estrogen receptor activation, cyclin D1 expression, and cell growth in prostate cancer, and antibodies to this protein develop in various malignancies. This suggests that CCDC62 may play a role in augmenting cellular viability, but its true role in PD awaits discovery. Huntingtin-interacting protein 1-related (HIP1R) is involved in actin binding and receptor-mediated endocytosis. Loss of function is associated with impaired presynaptic function and plasticity, leading to neuronal dysfunction. It appears to protect against polyglutamine toxicity in the transgenic *C. elegans* Huntington model.

Genes less strongly associated with PD risk include BDNF (11p13), the translation of which produces brain-derived neurotrophic factor. BDNF is critical to the survival of striatal neurons. A rare functional G196A (Val66Met) BDNF variant is associated with greater PD severity, earlier PD onset, and cognitive impairment. DRD3 (3q13.3) is the gene for the D3 dopamine receptor. Reduced lymphocyte DRD3 mRNA and the DRD3 2 allele are associated with PD. GSTM1 (1p13.3) encodes glutathione S-transferase mu 1 and is involved in detoxifying electrophilic compounds. The GSTM1 null genotype is linked to PD in the contexts of CYP2D6 poor metabolizer status and solvent exposure. PARK2 (parkin, 6q25.2-q27) mutations are classically associated with sporadic PD and with recessive, early-onset, slowly progressive, Lewy body-negative parkinsonism. Parkin is an E3 ubiquitin ligase enzyme of the ubiquitin-proteasome system, key to disposing of obsolete and toxic proteins. Additionally, parkin confers resistance to oxidative mitochondrial damage and to various apoptogenic stimuli. MAOB (Xp11.23) translation produces monoamine oxidase B. The MAOB G genotype is variably associated with reduced PD risk in Caucasian but not Asian men. CALB1 (8q21.3-q22.1) is the gene for the 28 kilo-Dalton calbindin 1. The CALB1 SNP rs1805874 is linked to PD risk through an unclear mechanism. LRP8 (1p34) is the gene for low-density lipoprotein receptor-related protein 8, associated with the apolipoprotein E receptor. LRP8 knockout increases tau phosphorylation in mice suggesting a relation to MAPT (see above). DRD2 (11q23) encodes the D2 dopamine receptor. Knockout in mice produces parkinsonism, and the TaqIa polymorphism, especially the A1A1 genotype, and 15-allele polymorphism are associated with PD motor fluctuation risk. DRD2-deficient mice manifest akinesia and bradykinesia resembling PD. PARK5 (UCHL1, 4p14) mutations are classically associated with PD onset in the 6th decade. UCHL1 is involved in maintaining ubiquitin monomers for proper functioning of the ubiquitin-proteasome system and has the weakest association with PD risk of the genes considered here. Figures 1 and 2 show the relationship of these genes to the pathobiological processes involved in PD.

For each drug, available findings for gene expression, replicated posttranslational findings (largely cell culture), and animal models are presented. The gene expression effects of psychotropics are considered for PD risk without regard to particular mutations, variants, and genotypes, which are beyond the scope of this paper. It is possible that reduced risk may also translate to slower PD progression, although correlates of risk and disease progression often differ. Replicated findings mostly involved cell culture, with the majority replicated *across* models (almost half of these *independently* replicated), and nearly half replicated *within* the same model (only valproate induction of *α*Syn was *independently* replicated). Independent replication within and without models was only evident for lithium and valproate, constituting the two most robustly replicated preclinical findings. Findings from animal models are then detailed. Most PD animal model studies of psychotropics have shown neuroprotective results, including pramipexole, lithium, valproate, lamotrigine, and dextromethorphan, in contrast to desipramine and fluoxetine, which actually shortened mouse survival.

Finally, following the presentation of transcriptomics, cell culture, and animal model findings, clinical trials of drugs constituting first-line psychotropics in human patients with PD are discussed.

### 3.1. Pramipexole

In the rotenone mouse model of PD, this dopamine D2/D3 receptor agonist decreased *α*Syn, neuronal death, and motor deficits [10]. In the MPTP rat model of PD, pramipexole inhibited reactive oxygen species (ROS) generation [11]. In the LPS rat model of PD, pramipexole preserved dopamine neurons and reduced ubiquitin upregulation and amphetamine-induced ipsiversive turning, but did not affect the inflammatory response [12]. In a 6-hydroxydopamine rat model of PD, pramipexole increased cell proliferation and survival, neural differentiation, neurogenesis, and epidermal growth factor mRNA in the subventricular zone and increased motor activity [13]. This drug has also increased both BDNF and glial-derived neurotrophic factor (GDNF) in mesencephalic and nigral astrocytic cell culture [14]. Each of these actions is consistent with a reduced risk of PD progression.

### 3.2. Ropinirole

Although this D2/D3 agonist has been demonstrated to be antiapoptotic in neuroblastoma cell lines, evidence in mature neural tissues was not evident. This drug, however, has been associated with increases in both BDNF and GDNF in rat mesencephalic cell and nigral astrocytic cultures, but not in striatal or cortical astrocytic culture [14]. In a study of mouse astrocytes taken from whole brain, ropinirole increased GDNF but not BDNF [15]. Ropinirole's neurotrophic effect on cultured mesencephalic dopamine neurons was inhibited by the D3 antagonist nafadotride [14]. These findings suggest neurorestorative effects of this drug.

### 3.3. Antipsychotics

Although it would be ideal to have studies conducted in blood and brain of patients with PD, no such studies have been reported, and the best data that can be obtained for the transcriptional effects of psychotropics has been determined in patients with psychiatric disorders. Chronic antipsychotic treatment downregulated LRP8 and UCHL1 (PARK 5) expression in schizophrenia [16, 17]. Antipsychotic administration downregulated ApoER2 (LRP8) mRNA in peripheral lymphocytes after 6 months of treatment compared to pretreatment baseline in drug-naive patients with schizophrenia [16]. In postmortem prefrontal cortex, chronic treatment was associated with downregulated UCHL1 mRNA relative to matched healthy controls and drug-naïve patients [17]. Since reductions in LRP8 and UCHL1 function are linked to PD, the effects of antipsychotics in these studies would be expected to increase PD risk and, possibly, PD progression (see Figure 2 for LRP8 and PARK5 relation to PD pathobiology).

### 3.4. Neuroleptics

Neuroleptic inhibition of mitochondrial respiratory Complex I in frontal cortex has been replicated [18, 19], suggesting an increased risk of PD progression and, perhaps, an increased risk of developing the disease.

### 3.5. Chlorpromazine

Six-month treatment with chlorpromazine upregulated prefrontal and temporal cortical DRD2 mRNA expression in primates [20], an effect that might reduce PD risk and progression.

### 3.6. Haloperidol

Haloperidol treatment is associated with DRD3, striatal PARK2 (parkin), and striatal DRD2 upregulation and nonstriatal BDNF downregulation. Four weeks of haloperidol induced striatal Park2 [21] and whole brain Drd3 [22, 23] expression in rats, suggesting parkin upregulation specific to the nigrostriatal system. In rat pituitary, 21 days of haloperidol upregulated D2 mRNA expression [24]. Although early striatal studies were negative in rodents [25–27], subsequent studies found upregulated striatal D2 mRNA expression changes after chronic haloperidol treatment [23, 28–32]. Four-week administration upregulated striatal and prefrontal cortical Drd2 expression in rats [22, 32]. In primates, 6-month treatment with haloperidol also upregulated prefrontal and temporal cortical D2 mRNA expression [20]. In contrast, haloperidol downregulated hippocampal and cortical Bdnf expression in rats [33–35], although one hippocampal study showed no change [36].

The replicated mitochondrial effects of haloperidol include Complex I inhibition in frontal cortex [18, 19], Complex II inhibition [37], and apoptosis-inducing factor (AIF) translocation [38].

In sum, while upregulation of striatal PARK2, DRD3, and DRD2 might reduce PD risk, BDNF downregulation, complex I and II inhibition, and AIF translocation would be expected to increase PD risk and could potentially predominate, increasing risk and perhaps progression (Figures 1 and 2). Of course, clinical exacerbation of parkinsonian neurological features effectively contraindicates the use of clinical doses of haloperidol in PD.

### 3.7. Loxapine

Loxapine administered for 32 days upregulated whole brain D3 [22, 23] and D2 [22, 32] mRNA in rats, thus suggesting an association of loxapine with PD risk reduction.

### 3.8. Molindone

Six-month treatment with molindone upregulated prefrontal and temporal cortical D2 mRNA expression in primates [20], a finding that is consistent with a potentially reduced PD risk.

### 3.9. Pimozide

Pimozide upregulated whole brain D3 mRNA in rats after 32 days [22] and upregulated prefrontal and temporal cortical D2 mRNA expression in primates after 6 months [20], suggesting a lowering of PD risk.

### 3.10. Risperidone

Risperidone [39, 40] treatment for 4 weeks in rats upregulated frontal cortical Maob expression while 6-month treatment upregulated prefrontal and temporal cortical D2 mRNA expression in primates [20]. The D2 result is consistent with a potentially reduced risk for PD. In contrast, risperidone inhibition of frontal cortical Complex I has been replicated, suggesting an increased risk of PD progression [18].

### 3.11. Olanzapine

Olanzapine upregulated hippocampal and cortical Bdnf [34], frontal cortical Gstm1 [9] and Maob [40], and ventral tegmental Drd2 [41] expression in rats, collectively indicative of reduced PD risk (Figures 1 and 2). Similarly, 6-month treatment with olanzapine upregulated prefrontal and temporal cortical D2 mRNA expression in primates, but in contrast to other drugs, olanzapine did not affect striatal DRD2 expression [20]. These findings nevertheless suggest a lower risk of PD, especially the tegmental finding.

### 3.12. Quetiapine

The rat literature reveals upregulated prefrontal cortical Bdnf mRNA with quetiapine [42], potentially consistent with reduced PD risk.

### 3.13. Clozapine

Although rat Bdnf studies reveal both upregulation [34] and downregulation [35] in the hippocampus and cortex with clozapine (10 mg/kg for 28 days) [34, 35], Drd3 expression was upregulated in whole brain after 32 days of treatment [22]. Six-month treatment upregulated prefrontal and temporal cortical D2 mRNA expression in primates, but in contrast to the other drugs, clozapine did not affect striatal DRD2 expression [20]. Nevertheless, clozapine inhibition of frontal cortical Complex I [18] and increase in Complex IV [19] have been replicated, likely indicating an increased risk of PD progression in light of reduced Complex I in PD (Figure 1). Therefore, it is unclear whether clozapine is associated with a reduced or increased risk of PD.

### 3.14. Aripiprazole

In the ventral tegmental area, aripiprazole increased D2 mRNA expression after 12 weeks of treatment [41], suggesting a reduced risk for PD.

### 3.15. Lithium

Lithium downregulated BDNF mRNA (while increasing BDNF itself) [43] and did not affect ventral tegmental D2 [44] mRNA expression in rats, suggesting neutral risk for PD. In contrast, replicated findings were confined to decreases in fibrillar tau in transgenic FTDP-17 models [45, 46] and cytochrome c release [47, 48], each associated with the likelihood of a neuroprotective reduced PD progression (Figure 1). In animal models, although in the G93A superoxide dismutase 1 mutant transgenic mouse model of ALS, lithium has been found to decrease both *α*Syn and ubiquitin aggregation [49]. Although in several different tauopathic FTDP-17 mouse models, lithium decreased tau phosphorylation at a variety of epitopes including Tau1 [45], Ser202 [50], AT8 [46], and PHF1 [46, 50] and decreased tau fibrillization [46] and fibrillar [45] and filamentous [45] tau aggregation. Human FTDP-17 has been particularly associated with Ser202 [51] and AT8 [52] phosphorylation. These models are not only relevant to FTDP-17, but potentially also to PD because tau and *α*Syn interact to mutually promote their production and aggregation, as explained below (see also Figure 2). Finally, lithium prevented nigrostriatal dopamine neuronal loss in MPTP mice [53]. Each of these findings is consistent with a reduced risk of progression in PD.

Effects on other proteins, such as A*β* and tau, are potentially important to the pathobiology of PD. A referenced discussion is beyond the scope of this paper; however, we have detailed the interactions of these proteins with *α*Syn elsewhere [4]. Briefly, A*β* and tau each facilitate *α*Syn aggregation in PD (Figure 2). *α*Syn also facilitates tau aggregation, and *α*Syn and tau each independently initiate amyloid formation, further facilitating *α*Syn aggregation. Furthermore, the enzyme glycogen synthase kinase 3 (GSK-3) promotes *α*Syn expression, A*β* production, tau phosphorylation, and apoptosis. GSK-3 alleles are associated with PD risk, and GSK-3 inhibitors including lithium and valproate may reduce *α*Syn. Moreover, *α*Syn upregulates GSK-3, suggesting that *α*Syn and GSK-3 mutually upregulate each other, and *α*Syn can indirectly upregulate A*β* and tau production and aggregation through this mechanism. Still further, each of these proteins (*α*Syn, A*β*, and tau) can interact at various levels in the pathological chain of events, leading to apoptotic pathway activation, neuronal death, and neuroinflammation. *α*Syn, A*β*, and tau each inhibit the proteasome, impair mitochondrial function, produce free radicals, and promote apoptosis. Thus, effects on tau and even A*β* can modulate PD pathobiology. In this regard, GSK-3 inhibitors, including lithium and valproate, can have potent effects on *α*Syn and, potentially, on PD pathobiology (Figure 2).

### 3.16. Carbamazepine

Carbamazepine upregulated BDNF mRNA expression in rat frontal cortex [54], suggesting a potentially neuroprotective reduced risk of PD.

### 3.17. Valproate

Replicated findings include increased *α*Syn [55–57] in several models including cell cultures exposed to 6-hydroxydopamine and glutamate [55, 56] and in the rotenone rat [57]. Valproate has inhibited apoptosis in glutamate [55] and rotenone [57] models. Valproate anti-apoptotically decreased monoubiquitylated *α*Syn [56, 57] and its nuclear translocation [56, 57] and inhibited free radical damage [58]. In the rotenone rat model of PD, valproate increased *α*Syn, decreased its apoptotic monoubiquitylation and nuclear translocation in both the substantia nigra and striatum, and prevented nigral apoptosis and nigrostriatal neuronal loss, as well as preventing the death and parkinsonian features observed in rotenone rats not treated with valproate [57]. In addition, valproate both protected and increased dopaminergic concentrations in rat mesencephalic mixed neuronal-glial cell cultures after exposures to either LPS or MPTP [59]. Furthermore, valproate increased BDNF and GDNF transcription in astrocytic cell cultures [59, 60]. These actions are consistent with neuroprotection, neuroregeneration, and a reduced likelihood of PD progression (Figures 1 and 2).

It is noteworthy that, like lithium, valproate too is a GSK-3 inhibitor. It can therefore potentially produce potent therapeutic effects on PD pathobiology through attenuation of *α*Syn, A*β*, and tau proteins, as detailed in the section on Lithium (see also Figure 2). Moreover, valproate is also a histone H3 deacetylase inhibitor (H3 HDACI), and this action has been correlated with neuroprotective increases in *α*Syn levels [61], possibly upregulating the expression of other risk-attenuating genes while interfering with the repression of risk-associated genes [57]. (Although the role of *α*Syn in neurodegeneration has been extensively debated and excessive *α*Syn would seem to predispose to the formation of Lewy bodies that are associated with PD, recent evidence suggests a neuroprotective function of the protein and that it is the depletion of *α*Syn concentrations and conversion to a monoubiquitylated species traveling to the nucleus that instead promotes *α*Syn fibrillization and neurodegeneration [57]). Additionally, H3 HDACIs including valproate and dextromethorphan (see Dextromethorphan section) are protective in MPTP and LPS models and appear to protect dopamine neurons by upregulating astrocytic GDNF and BDNF. H3 HDACIs further induce microglial apoptosis (thereby reducing microglial neuroinflammation) and attenuate LPS-induced dopaminergic neurotoxicity [62], again like dextromethorphan. Sirtuins are members of the histone deacetylase family, and inhibition of sirtuin 2 protects cells in PD models [63]. Nuclear *α*Syn has been shown to inhibit histone acetylation, leading to cellular demise [64], while HDACIs mediate the opposite action. Valproate's GSK-3 and H3 HDACI properties may factor into a neuroprotective effect in PD in a significant way (Figures 1 and 2).

### 3.18. Lamotrigine

This anticonvulsant has been confirmed to reduce striatal lesions by almost 50% in the MPTP rat model of PD, with an additional 14% reduction when coadministered with CoQ10 [65], consistent with a neuroprotective action and reduced risk of PD progression.

### 3.19. Antidepressants

Findings replicated across antidepressants include neuroprotective decreases in inflammatory cytokine expression, including IL-1*β*, IL-6, and TNF-*α* [66, 67]; however these same drugs (desipramine and fluoxetine) also have reduced survival in MPTP mice [68]. An overall neuroprotective profile for a number of antidepressants suggests that reduced survival may be unique to the MPTP model and that the antidepressants hold the potential to reduce PD progression.

### 3.20. Amitriptyline

Amitriptyline treatment for at least 21 days in rats upregulated nucleus accumbens shell D3 and striatal D2 mRNA expression [69], consistent with reduced PD risk.

### 3.21. Imipramine

In rats treated for at least 21 days, imipramine upregulated hippocampal Bdnf [70, 71], nucleus accumbens Drd3 [69], and striatal Drd2 [69] expression, indicative of reduced risk for PD (Figure 1).

### 3.22. Desipramine

Desipramine given for 21 days upregulated Bdnf in rat hippocampus [43, 72–74] and frontal cortex [74] but, in two studies, downregulated hippocampal mRNA [75] and had no effect on cortex [43, 75]. Treatment for at least 21 days upregulated nucleus accumbens shell D3 and striatal D2 mRNA expression [69]. These findings suggest reduced PD risk with desipramine. Further, decreased neuronal apoptosis in the context of desipramine treatment has been replicated [76, 77], suggesting a neuroprotective diminished risk of PD progression. On the other hand, desipramine treatment in a MPTP mouse model resulted in diminished animal survival [68], perhaps unique to this particular model.

### 3.23. Nortriptyline

The replication of the finding that this tricyclic antidepressant decreased neuronal apoptosis [76, 78] indicates a neuroprotective potential to reduce PD progression.

### 3.24. Fluoxetine

Fluoxetine administered for 21 days upregulated Gba, Ccdc62, Hip1R, Bndf, and Uchl1 and downregulated Mapt, Mccc1, Gstm1, and Calb1 expression in rat frontal cortex [8]. In other rat studies of this antidepressant, this same treatment course upregulated Bndf mRNA in frontal cortex and hippocampus [74, 79] (although without effect in one hippocampal study [75]) and in ventral tegmental area and nucleus accumbens shell but not in substantia nigra or striatum [79]. Fluoxetine treatment for at least 21 days upregulated nucleus accumbens shell Drd3 but not striatal Drd2 expression in rats [69]. Overall, the findings suggest a reduced risk of PD (Mapt, Gba, Ccdc62, Hip1R, Bdnf, Drd3, Uchl1) that likely predominates over risk-enhancing effects (Mccc1, Gstm1, Calb1) (Figures 1 and 2), although not specific to the nigrostriatum.

In the MPTP model of PD, however, fluoxetine actually reduced mouse survival [68]. Whether this is unique to this model or will generalize across PD models remains to be determined.

### 3.25. Sertraline

In rats treated for 21 days, sertraline upregulated BDNF mRNA expression [72], consistent with a reduction in PD risk.

### 3.26. Paroxetine

Paroxetine administered for 21 days upregulated Bdnf expression [80], consistent with a lower risk for PD.

### 3.27. Escitalopram

In patients with depression, escitalopram treatment for 12 weeks increased leukocyte BDNF mRNA expression, correlating with serum BDNF level [81] and suggestive of reduced risk for PD.

### 3.28. Venlafaxine

Venlafaxine upregulated Bdnf expression in rats treated for 21 days [71], suggesting a lowering of risk for PD.

### 3.29. Duloxetine

In rats treated for 21 days, duloxetine upregulated BDNF mRNA [82, 83], suggestive of a lower risk for developing PD.

### 3.30. Bupropion

Chronic bupropion downregulated hippocampal expression of Bdnf [75], consistent with an increase in PD risk.

### 3.31. Diazepam

Decreased cytochrome c release after treatment with diazepam in neurons exposed to t-butyl-hydroxy-peroxide [84] has been replicated across models and suggests a dose-dependent neuroprotective reduced risk of PD progression. However, higher doses have promoted apoptosis in other models (see [3] tables published online on journal website).

### 3.32. Dextromethorphan-Quinidine Combination

Replicated findings in both MPTP and neuroinflammatory LPS models of PD for dextromethorphan include decreased midbrain dopaminergic neuron degeneration in rat mesencephalic cell culture [85, 86] and protection of dopamine concentration, dopamine neurons, and locomotor activity in mice [87].

In MPTP mice, dextromethorphan protected dopamine neurons [87, 88], dopamine concentrations [87], and locomotor activity [87] and reduced glutamatergic excitotoxicity on dopamine neurons [89]. A previous study had not demonstrated protection of dopamine concentrations in this model [89]. Dextromethorphan also protected dopamine concentrations in mice treated with both MPTP and diethyldithiocarbamate [89]. In the methamphetamine mouse model of PD, dextromethorphan protected dopamine neurons and prevented microglial activation [90]. Finally, in the mouse neuroinflammatory LPS model of PD, dextromethorphan protected dopamine neurons, dopamine concentrations, and locomotor activity [87].

Similarly, replicated findings for the dextromethorphan metabolite 3-hydroxymorphinan (3-OHM) include decreased dopamine neurotoxicity in rat mesencephalic cell culture [87, 91] and protection of dopamine neurons, dopamine concentrations, and locomotor activity [87] in MPTP and LPS mouse models. 3-OHM was even more potently protective than dextromethorphan in both models, an effect that was mediated by enhanced astroglial neurotrophic effects and attenuated microglial activation [87].

Dextromethorphan and its 3-OHM metabolite may protect dopaminergic neurons by decreasing neuroinflammation related to microglial activation with its attendant increases in ROS, reactive nitrogen species, and TNF-*α*, and also by increasing astrocytic neurotrophic support [85–88, 90, 91] (Figure 1). Additionally, dextromethorphan may protect dopaminergic neurons by blocking glutamate excitotoxicity [89] (Figure 2). Furthermore, the 3-hydroxy metabolite has been found to increase histone H3 acetylation (like valproate [55]) and neurotrophins including GDNF and several others (like valproate (see Valproate section) and antidepressants [4]) [87] (Figure 1). GDNF and BDNF have demonstrated neuroprotection of nigrostriatal neurons in several PD models, with GDNF being even more potent than BDNF [4].

Although there were no transcriptomic data available for dextromethorphan/quinidine, it is interesting to consider whether dextromethorphan/quinidine is inadvisable in GSTM1 null genotype patients, since this genotype is associated with PD risk in the context of CYP2D6 poor metabolizer status, and quinidine inhibits CYP2D6. This same concern might also apply to other CYP2D6 inhibitors, including the psychotropics haloperidol, fluoxetine, paroxetine, duloxetine, and bupropion.

### 3.33. Neuroprotective Clinical Trials

Several studies have attempted to look at markers that can potentially ascertain neuroprotective disease-modifying outcomes. These include clinical trials of ropinirole, pramipexole, and dextromethorphan. Results have been inconclusive to date.

Dopamine agonists constitute first-line neuropsychiatric treatments for apathy and are a mainstay of treatment for PD. Several double-blind parallel group clinical trials have considered course-of-illness slope divergence to ascertain neuroprotective properties in PD. These studies also employed positron emission tomography (PET) and include a 5-year multicenter study of 288 patients with early PD randomized to either ropinirole or L-DOPA [92], the REAL-PET 2-year multicenter trial in 186 patients with PD randomized to ropinirole or L-DOPA [93, 94], a study of 45 patients randomized to ropinirole or L-DOPA [95], and the CALM-PD 2-year multicenter trial in 301 patients with PD randomized to either pramipexole-plus-placebo “L-DOPA” or L-DOPA-plus-placebo “pramipexole” [96]. The ropinirole study involving 288 patients found less dyskinesia with ropinirole but no difference in clinical markers of PD progression [92], and it is not clear that dyskinesia can be considered as a marker of PD pathobiological progression. The ropinirole trial in 45 patients revealed no significant differences between ropinirole and L-DOPA in terms of 18F-dopa uptake deterioration (13% in 28 versus 18% in 9 patients) at 2 years compared to baseline [95]. The CALM-PD study showed less dopaminergic motor complications with pramipexole but greater UPDRS Parkinson scale improvement with L-DOPA or L-DOPA-plus-placebo “pramipexole” [96], yet neuroprotective conclusions are not possible because of uncertain dose equivalence between the two study arms and other limitations. Imaging markers in the REAL-PD investigation revealed slower putamenal (18)F-DOPA (dopaminergic presynaptic terminal marker) signal decline with ropinirole [93, 94] while the CALM-PD study demonstrated reduced *β*-CIT (dopamine transporter marker) decrement with pramipexole [97]; however alternative pharmacological explanations [98, 99] and other limitations preventing neuroprotective conclusions for the REAL-PET [98, 100] and CALM-PD [99, 101] studies have been detailed.

Dextromethorphan combined with quinidine is a new FDA-approved treatment for pseudobulbar affect. In small clinical trials, dextromethorphan (alone, without quinidine) has improved PD signs in two studies [102, 103] and improved dyskinesia and off time in two others [104, 105], although PD signs did not improve in another study employing a lower dose [106]. None of these studies were designed to assess neuroprotection.

Hence, currently, there is no conclusive clinical evidence of disease-modifying neuroprotection for psychotropics although the clinical trial literature in PD is miniscule.

## 4. Conclusion

Preclinical findings for the specific drugs are summarized in Table 1. Transcriptional effects are subject to the caveats described below. MAPT, GBA, BDNF, and DRD2 genes have the clearest relations to PD risk based on knockout models, null alleles, mutation severity correlations, and haplotype analysis. MAPT, GBA, MCCC1, CCDC62, and HIP1R are most strongly linked to PD in risk association studies. These data indicate that downregulation of MAPT and upregulation of GBA, CCDC62, HIP1R, and perhaps BDNF and DRD2 may reduce PD risk, reflected in Table 1, whereas the effects of transcription regulation of the other genes on PD risk are more tentative. Other preclinical finding caveats are detailed below.

The findings above provide an index of the neuroprotective potential of psychotropics in PD. Fluoxetine had salutary transcriptional effects on 7 of the 10 risk genes studied although its effect on posttranscriptional events is wanting, and it shortened mouse survival in an MPTP model. Drugs with multiple actions that may confer disease-modifying neuroprotection include dextromethorphan, valproate, lithium, and pramipexole. These drugs have neuroprotective effects on *α*Syn, except that the HDACI dextromethorphan lacked direct data for this protein, and lithium had neuroprotective effects on both *α*Syn and tau protein. One potential therapeutic strategy that might be tested in animal models and humans is the combination of valproate with dextromethorphan in attempting to therapeutically modulate H3 HDAC, GSK-3, *α*Syn, ROS, apoptosis, and trophic factors. Desipramine (transcriptional and antiapoptotic properties) and escitalopram (transcriptional and trophic attributes) might also be worth considering.

In general, most drugs other than bupropion and lithium had beneficial transcriptional effects. This benefit would not necessarily extend to patients with certain gene variants or mutations, where these transcription effects might actually increase PD risk. Most antipsychotics inhibited Complex I, which is already robustly inhibited in PD. Only pramipexole, valproate, and dextromethorphan demonstrated replicated attenuation of ROS while only valproate, desipramine, and nortriptyline showed consistent replicated antiapoptotic activity, although desipramine curiously shortened survival in the MPTP mouse. Whether this result will be obtained in other PD models remains to be elucidated. Pramipexole, valproate, and dextromethorphan have shown replicated protective effects in LPS inflammatory models, although anti-inflammatory mechanisms await replication while a neurotrophic mechanism has been documented [59]. Pramipexole, ropinirole, and valproate have demonstrated replicated increases in both BDNF and GDNF whereas escitalopram increases BDNF and dextromethorphan increases GDNF.

Enthusiasm for applying the transcriptional results must be tempered by limitations including variable PD-risk associations of these genes in different populations, changing gene definitions and gene risk rankings over time, variable effects depending on treatment durations, brain region, and stage of illness, multiple transcriptional effects of drugs with sometimes contradictory risk effects (e.g., fluoxetine), rodent-human translational issues, an incomplete understanding of gene roles in PD pathogenesis, and the uncertainty of how much of the variance in clinical neuroprotection might be accounted for by transcriptional effects. The effects of psychotropics on the expression of these genes should now be studied using RT-PCR, particularly in the substantia nigra and striatum.

There are several caveats in interpreting how well the replicated findings can generalize to and predict clinical translation. These include limitations inherent to a literature review, reporting biases, uneven and unsystematic drug investigation across the various actions of interest, varying predictive validities of PD animal models, and varying drug effects that can depend on dose, treatment duration, apoptogen, neurotoxin, additional disease-modifying mechanisms of action, and stage of illness. A given psychotropic can possess plural neuroprotective and prodegenerative effects and can act simultaneously as both friend and foe, depending on the relative weights of these effects. Additionally, increases in *α*Syn alone can be either neuroprotective or prodegenerative, depending upon context. An increase in *α*Syn can lead to proteasomal inhibition, apoptosis, and inclusion formations including Lewy bodies, pathological tau, and A*β*, and yet a rise in *α*Syn can also effect a neuroprotective response. It appears that monoubiquitylation of *α*Syn with subsequent translocation to the nucleus may engender apoptosis, in contrast to an otherwise neuroprotective rise in non-monoubiquitylated *α*Syn [57]. In this regard, evidence that valproate decreased *α*Syn monoubiquitylation and nuclear translocation in both the substantia nigra and striatum of the rotenone rat is tantalizing [57].

At present, no disease-modifying neuroprotective agents have been conclusively demonstrated to be effective in human clinical trials. These studies have relied on a difference in slope deterioration between treatments to measure progression, rather than the use of neuroprotective paradigms. Clinical trials employing delayed-start or randomized-withdrawal designs [107] are needed to resolve the neuroprotective disease-modifying efficacy of ropinirole, pramipexole, and dextromethorphan in PD. These randomized designs assess disease-modifying effects by comparing two active treatment arms to each other, with the comparator arm receiving placebo for a protracted period followed by active treatment initiation after a delayed period (delayed start), or a switch from active treatment to placebo in the comparator arm substantially before the continued treatment arm is completed (randomized withdrawal). In this way, differences in outcome as a function of differing treatment durations can be assessed. Similarly, studies should be undertaken for the other promising psychotropics that exhibit salutary effects in animal models and have replicated *in vitro* findings. It will be interesting to learn the results of neuroprotective trials for these commonly used treatments in patients with PD.

## Figures and Tables

**Table 1:** Preclinical effects of psychotropics on PD pathobiology.

	Gene	Protein	Psome	Cmplx	Mt	ROS	Apop	Inflam	Trophins	Animal
Pramipexole		+ *α*Syn				+			+	+
Ropinirole									+	
Antipsychotics	−									
Neuroleptics	+			−						
Chlorpromazine	+									
Haloperidol	+			−	−					
Loxapine	+									
Molindone	+									
Pimozide	+									
Risperidone	+			−						
Olanzapine	+									
Quetiapine	+									
Clozapine	+			−						
Aripiprazole	+									
Lithium	0	+tau, *α*Syn								+
Carbamazepine	+									
Valproate		+*α*Syn				+	+		+	+
Lamotrigine										+
Antidepressants	+									−
Amitriptyline	+									
Imipramine	+									
Desipramine	+						+			−
Nortriptyline							+			
Fluoxetine	+									−
Sertraline	+									
Paroxetine	+									
Escitalopram	+								+	
Venlafaxine	+									
Duloxetine	+									
Bupropion	−									
Diazepam							+			
Dextromethorphan						+			+	+

The effect of psychotropics on PD pathobiology is indicated by a “+” indicating actions consistent with reducing PD risks of onset (gene transcription effects) or progression (other actions). “−” represents actions that are consistent with enhancing risks of onset or progression. “0” indicates neutral risk: *Psome:* proteasome; *Cmplx:* mitochondrial respiratory chain complexes; *Mt:* mitochondrion, *ROS:* reactive oxygen species, *Apop:* apoptosis, *Inflam:* inflammation.
